# Evaluating the disaster preparedness of emergency medical services (EMS) facilities: a cross-sectional investigation in Iran

**DOI:** 10.1186/s12873-024-00932-z

**Published:** 2024-03-25

**Authors:** Mehdi Beyrami Jam, Mohsen Aminizadeh, Yousof Akbari-Shahrestanaki, Hamid Reza Khankeh

**Affiliations:** 1 Social Determinants of Health Research Center, Research Institute for Prevention of Non-Communicable Diseases, Qazvin University of Medical Sciences, Qazvin, Iran; 2 Health in Disasters and Emergencies Research Center, Institute for Futures Studies in Health, Kerman University of Medical Sciences, Kerman, Iran; 3 Department of Pre-Hospital Medical Emergencies, School of Paramedical, Qazvin University of Medical Sciences, Qazvin, Iran; 4 Health in Emergency and Disaster Research Center, University of Social Welfare and Rehabilitation Sciences, Tehran, Iran

**Keywords:** Emergency medical services, Emergencies, Disasters, Hazard analysis, Preparedness

## Abstract

**Introduction:**

The preparedness of Emergency Medical Services (EMS) plays a crucial role in managing health disaster risks. This study was conducted to assess the disaster preparedness of EMS facilities in Iran, using a nationally reliable tool.

**Methods:**

A cross-sectional study was conducted in May 2021 to evaluate the disaster preparedness of EMS facilities in Iran using a national standard tool. The census sampling approach was utilized to select the samples, and descriptive statistics, as well as analytical statistics like the independent t-test and Pearson’s correlation test, were used to analyze the data using SPSS-18 software.

**Results:**

The findings of this study revealed that the majority of EMS facilities in Iran possess a moderate level of preparedness in dealing with disasters. Also, the results indicate that EMS facilities have an average level of preparedness against disasters in all dimensions except for “coordination and cooperation” and “surge capacity”.

**Conclusion:**

According to this study, the majority of EMS facilities in Iran are exposed to different disasters. Furthermore, the findings indicate that while EMS facilities are generally prepared to respond to disasters at an average level, there are some important preparedness gaps in dimensions like response capacities, coordination, and cooperation. Thus, specific strategies, standards, and procedures must be developed and disseminated by the National Medical Emergency Organization.

## Introduction

As an integral part of healthcare systems, emergency medical services (EMS) are at the frontline of the healthcare systems during emergencies, disasters, and life-threatening injuries [1]. In many countries, EMS is a “gatekeeper” to access specialized hospitals in emergencies and disasters [2]. Emergency medical services, also known as ambulance services, are comprehensive emergency services in the community that organize personnel, facilities, and equipment to provide urgent, effective, and well-coordinated pre-hospital care to individuals experiencing an unexpected injury or illness and to transport them to medical facilities [3].

Natural and man-made disasters have destructive effects on human life and the environment. They cause deaths, injuries, and disruptions in economic and social functions in the affected areas [4–6]. The latest statistics released globally (2022) indicate that in the past decade, an average of 45,000 people have died annually due to disasters [4]. Only in 2022, 14,442 deaths due to natural disasters have been recorded in the world [4].

Due to its geographical location, Iran is considered one of the most disaster-prone countries in Asia and the world [7]. A total of 249 natural disasters such as floods, earthquakes, landslides, avalanches, etc. have been recorded in different regions of the country from 1900 to 2021 [2, 8]. In addition to natural disasters, man-made disasters such as traffic accidents, plane crashes, etc. also always happen [8].

The World Health Organization (WHO) considers disasters as a situation where local equipment and tools necessary to support human life have been destroyed due to natural or man-made disasters [2]. Given the high frequency of disasters, their management and controlling complications are necessary to preserve societies and ensure their stability [2].

The healthcare system in any country can apply its management in a time of disaster if it manages to control the incident from the beginning and at the scene [2]. This requires effective management of the incident from the pre-hospital phase. As much as the EMS system is well trained, competent, and in other words, more prepared, it will be effective in reducing casualties and deaths in disasters under any scenario [9–11]. In the world, there is a consensus on EMS coordination, efficiency, and preparedness in response to disasters. Thus, the preparedness of EMS plays a vital role in disasters [11].

The conventional perception of preparedness is that it consists of actions meant to strengthen coping mechanisms and response skills [10, 12]. According to the United Nations Office for Disaster Risk Reduction (UNISDR) disaster preparedness is "the skills and abilities that governments, response and recovery groups, communities, and people have developed to effectively prepare for, respond to, and recover from the effects of probable, recent, or impending disasters" [13]. According to Beyamijam et al. (2021), following codified laws and legal requirements, EMS disaster preparedness is a dynamic, ongoing, activity-based, and context-dependent process that through the establishment of management structures, capacity building, communication development, improved coordination and cooperation, as well as exercising and training, improves disaster response capabilities [14]. In recent studies, various components have been mentioned for disaster preparedness [15–19]. According to these investigations, key elements in disaster preparedness include building capacity, utilizing information and communication technology, planning for disasters, effective communication, training for emergencies, policy development, financial backing, coordination efforts, provision of safety and security, implementation of early warning systems, experience in disaster response, and legal considerations [15–19].

To the best of our knowledge, a small number of studies have been carried out to evaluate EMS disaster preparedness in Iran and other countries. The results of a review study indicate that the majority of the research on this topic was conducted in the United States and many of the EMS agencies in the world are not well prepared to respond to disasters [18]. Additionally, this study demonstrated the majority of the selected studies in this review have employed non-standard researcher-made instruments that were not subjected to the psychometric procedure during their development [18]. Therefore, the current study aimed to investigate the disaster preparedness of EMS facilities in Iran using a national standard tool and an all-hazards approach.

## Methods

### Study design and settings

This cross-sectional study, conducted in a descriptive-analytical manner at Qazvin University of Medical Sciences, aimed to assess the disaster preparedness of EMS facilities affiliated with universities of medical sciences in Iran.

### Populations, sampling, inclusion, and exclusion criteria

In keeping with the objectives of the study, we chose all of Iran's EMS facilities using a census sample technique. The eligibility criteria included the willingness and agreement of the highest authority of the EMS facilities to participate in the study and provide the necessary permissions to collect data and complete the research instrument. The exclusion criteria included the instrument's incomplete completion and failure to finish and submit it within the allotted time.

### Instruments

This study used a national(Iranian) tool- developed and validated by Beyramijam et al. (2021)- for evaluating the disaster preparedness of EMS facilities [14]. This tool measures EMS disaster preparedness with an all-hazard approach. The validity of the tool was assessed as 0.89 by surveying 15 EMS specialists and disaster risk management professionals. To determine the tool's reliability, ten EMS facilities' disaster preparedness was examined by two assessors, and the outcomes were evaluated using the inter-rater reliability approach and the reliability of the tool was estimated at 0.98. This tool contains two sections: The first section measures the demographic data and the second section contains items that measure the disaster preparedness of EMS facility. The demographic items measure the expertise and work history of the manager of the EMS facility, his/her attendance in disaster risk management (DRM) and training courses, the performance of disaster maneuvers in the EMS facility during the last year, and 5 hazards with the top priority in the EMS territory. The second section comprises 81 items with three choices: "not at all (score = 0)," "to some extent (score = 1)," and "absolutely (score = 2)." These items are categorized into 6 main domains and 18 sub-domains, assessing the disaster preparedness of EMS facilities. The “not at all” option means that no action specified in the relevant item has been carried out, or that it has only been developed and not implemented. The option “to some extent” means only a part of the actions mentioned in the item has been carried out, or only a part of the resources is available. Finally, the option “absolutely” indicates that all the necessary actions detailed in the item have been completed thoroughly. The main areas measured by the tool are the development of management-leadership structures (22 items), communication (9 items), coordination and cooperation (6 items), surge capacity (22 items), safety and security (10 items), and training and exercise(drill) (12 items). The items in the tool were rated by standardizing the obtained scores. To do so, the overall scores and areas of the tool are first converted into percentages with three sub-domains: 0–33% = poor preparedness, 34–67% = moderate preparedness, and 68–100% = strong preparedness. Before starting the data collection process, the head of Iran's National Medical Emergency Organization was informed about the study and its goals, and the required permissions were obtained. Next, the tool, its guide, and a letter of approval from the head of the National Medical Emergency Organization were sent to all EMS facilities in Iran in May 2021. They were instructed to complete the instrument within three months, using the available documents and disaster preparedness measures that were done following the goals and research questions. To provide further instructions on how to complete the tool and respond to inquiries, the researchers of the study maintained continuous phone and online communication with representatives of the EMS centers (mainly, the director of the disaster Management Unit) during this time. Following completion, the instruments were added to and gathered within the National Medical Emergency Organization's portal system. Ultimately, descriptive statistics like mean and standard deviation as well as analytical tests like Pearson's correlation test and independent t-test were used to evaluate the data using SPSS-18 software.

## Results

**Fig. 1:**
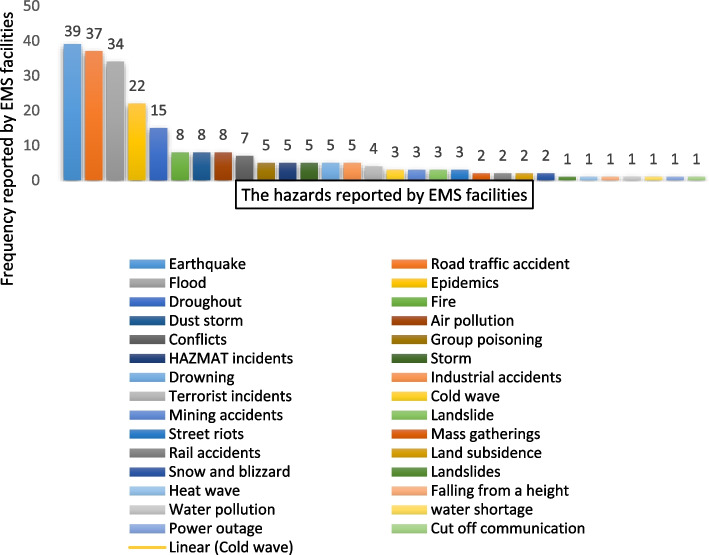
The most frequent hazards reported by EMS facilities

Out of 56 EMS facilities, 52 centers completed the questionnaire (a 92% response rate). A total of 31 hazards were reported by EMS facilities. The most frequent hazards were earthquakes, traffic accidents, floods, and epidemics (Fig. 1). Most of the managers of the EMS facilities were general practitioners (44.3%) and emergency medicine doctors (23.1%) (Table 1). An assessment of the disaster preparedness scores indicated that most of the EMS facilities (59%) in Iran had an average preparedness level (67.29 ± 27.35), 34% of the facilities had strong preparedness, and 5.79% had weak preparedness (Table 2). The data also indicated that the EMS facilities had a moderate level of preparedness in all disaster preparedness domains except for training and practice (that was at a favorable level). The preparedness levels of the EMS facilities were assessed to be 62.77%, 64.94%, 54.83%, 53.6%, and 61.35% in terms of management and leadership, communication, coordination and cooperation, surge capacity, and safety and security, respectively (Table 2). Furthermore, a comparison of the preparedness scores in all assessed areas showed that most EMS facilities had weak preparedness in terms of “coordination and cooperation” and “surge capacity” and had strong preparedness in terms of “training and practice” and “communication” (Table 2). The impact of the attendance of the EMS managers in DRM training courses on disaster preparedness of EMS facilities was assessed using the independent samples t-test. The results showed a significant difference between the mean preparedness score (106 ± 24) of the EMS facilities whose managers had attended the DRM training courses and the mean preparedness score (80.57 ± 27) of the centers whose managers had not attended DRM courses (*P* = 0.002). A significant difference was also observed between the mean preparedness score of the centers that had held training courses (drills) during the last year (103 ± 25.14) and the mean preparedness score of the centers that did not have any drills and practice (62 ± 27.71) during the last year (*P* = 0.001). However, Pearson’s correlation test did not show a significant correlation between the number of drills held by the EMS center in a recent year and the center’s overall preparedness score (r = 0.270; *n* = 52; *P* = 0.061).

**Table 1:** Demographic and professional information on EMS facilities

Variables	Categories	Frequency	Percentage
Gender	Female	0	0
	Male	52	100
Work experience as an EMS manager	1–5 yrs	39	75
	6–10 yrs	11	21.15
	10–15 yrs	2	3.84
Educational degree of EMS manager	Bachelor’s degree	9	17.3
	Master’s degree	1	1.9
	Medical doctor	23	44.2
	Emergency medicine	12	23.1
	Ph.D	4	7.7
EMS manager’s attendance in DRM training courses	Yes	38	73.1
	No	14	26.9
Doing drills in the past year	Yes	44	84.6
	No	6	11.53

**Table 2:**
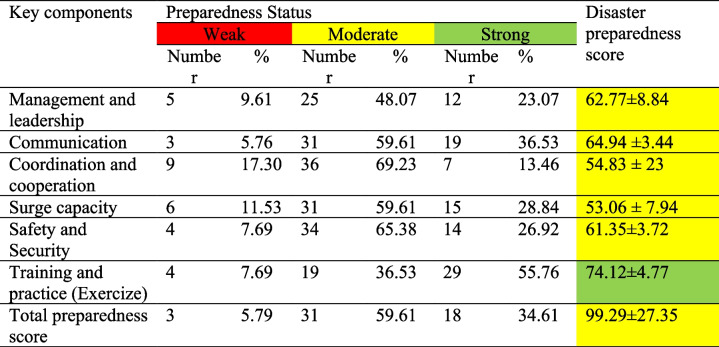
Average disaster preparedness scores for 6 key components of research tool at 52 EMS facilities

## Discussion

Identification of 31 hazards by pre-hospital emergency centers shows that Iran is a disaster-prone country. The findings from the present study showed that hazards such as earthquakes, road traffic accidents, floods, epidemics, and droughts are among the top five hazards reported by most EMS facilities. Since the present study was conducted during the COVID-19 outbreak in Iran, it was expected that the risk of the spread of epidemic diseases would be one of the important hazards for many EMS facilities. In addition to the hazards identified in this study, EMS facilities were also exposed to other hazards that were less serious compared to the five main hazards detailed above. Similarly, Khankeh et al. (2021) [20] introduced a hazard assessment tool and identified the first top 20 hazards rated by Iranian universities of medical sciences. They reported road traffic accidents, earthquakes, droughts, floods, and epidemics to be the five most serious health hazards. The authors identified 61 hazards in the first phase of the study. Afterward, they extracted 20 hazards with high priority in the healthcare system.

The data in the present study indicated that the EMS facilities in Iran have a moderate level related to disaster preparedness. The findings indicated that the level of preparedness for a specific natural or man-made hazard can be different. In a similar vein, Jadidi et al. (2019) investigated the level of preparedness of EMS facilities in 31 provinces of Iran in the face of Ebola. The results showed that the EMS facilities in Iran were highly prepared for response to Ebola [21]. Studies conducted in other countries have indicated that EMS is not adequately prepared to respond to disasters. For instance, Alotaibi et al. (2019) found that the EMS system of Saudi Arabia has limited preparedness in response to high mass casualty incidents [22]. Furthermore, Jama et al. (2016) [23] found that the Finnish emergency medical services are not sufficiently prepared to respond to and manage chemical emergencies. Maguire et al. (2007) [24] also reported that most EMS agencies in the United States do not have formal and extensive plans to respond to bioterrorism attacks and widespread epidemics.

The present study also examined the core dimensions of disaster preparedness in EMS facilities. The data showed that the EMS facilities had poor performance in terms of coordination-cooperation, and surge capacity dimensions. In this regard, the study of Sorani et al. (2018) showed that information management systems, and particularly the absence of integrated hospital information systems, were among the main challenges that the emergency medical system (EMS) faced following a disaster [25]. In this study, key elements contributing to a decline in the preparedness score of EMS facilities in the "coordination and cooperation" dimension, included insufficient planning and the absence of coordinated joint training sessions and exercises with other relevant organizations. Additionally, a deficiency in sharing information regarding operational plans, equipment, and supplies was observed. Enhancing conditions in these specific situations appears to be crucial for enhancing the preparedness of EMS facilities in the face of disasters. Moreover, membership in the High Disaster Committee or Council, coordination and close working relationships both inside and outside the organization before and during disasters, and interaction between local and provincial (state) EMS facilities are some of the activities and strategies highlighted in the literature to improve the disaster preparedness of EMS in the coordination dimension [21, 24].

The results of this study showed that at least 11% of the EMS facilities need more effort to improve their preparedness in the "surge capacity" dimension. In this regard, in the study of Sorani et al. (2018), the lack of adequate communication equipment, lack of trained manpower, and the lack of sufficient equipment to evacuate a large number of casualties were the main challenges of the EMS system in disasters [25]. In the current study, the absence of a strategy or instructions to address the well-being requirements of employees, volunteers, and their families, the lack of a procedure to monitor and handle the mental well-being of employees, volunteers, and their families, and also, deficiency in appreciating the contributions of employees and their families in disasters were the significant factors leading to a decline in the preparedness score within the human resource as a sub-dimension of the surge capacity dimension. Regarding equipment and logistics, which is a sub-dimension of surge capacity, the lack of special ambulances for patients in need of respiratory isolation (the ambulance equipped with High-efficiency particulate air filters(HEPA)), as well as the lack of necessary equipment to respond to CBRN incidents, including personal protective equipment( PPE) and decontamination equipment, were among the most important issues that most EMS facilities had mentioned. Therefore, it is expected that EMS officials will increase the disaster preparedness of EMS facilities by providing these types of equipment. The literature suggests that improving surge capacity can be achieved by creating plans and strategies to expand the response capacity in financial resources, physical spaces and structures, medical and logistic equipment, and human resources. literature have suggested ways to address the "surge capacity" dimension, including establishing and allocating EMS centers according to population density, recruiting enough skilled personnel(staff), and supplying medical and logistical supplies(stuff) as well as personal protective equipment in accordance with potential risks and circumstances [21, 24, 26].

The findings from the present study also indicated that more than half of the EMS facilities had a good performance in terms of “training and practice”. Training for pre-hospital emergency service providers is a critical element of disaster preparedness. The evidence from previous studies suggests that planning and implementing DRM training programs improves the perception of disaster preparedness [2, 27, 28]. In the current study, 73% of EMS managers have participated in DRM training courses. Furthermore, the organization and execution of disaster drills by a significant 84% of EMS facilities have proven crucial in enhancing the disaster preparedness situation within the training and practice dimension. The observations made in the present study revealed that organizing and holding training courses and the attendance of the manager of the EMS in DRM training courses can improve the disaster preparedness of EMS facilities in Iran.

### Limitations of the study

Since this study was conducted during the COVID-19 outbreak and extensive social distancing constraints, the researchers were unable to physically visit the EMS facilities to review documents and face-to-face interviews. Therefore, the data were collected by the director of the disaster management unit of EMS facilities using self-report instruments. It is recommended that future studies assess the disaster preparedness of EMS facilities in Iran through in-person evaluations.

## Conclusion

According to this study, the majority of emergency medical services (EMS) facilities in Iran are vulnerable to natural disasters such as floods, earthquakes, traffic accidents, droughts, and epidemics. As a result, EMS centers should establish the appropriate strategies to enhance their preparedness and response to disasters. Furthermore, the findings indicate that while EMS facilities are generally prepared to respond to emergencies and disasters at an average level, there are some important preparedness gaps in areas like response capacities and coordination and cooperation, particularly extra-organizational coordination. Specific strategies, standards, and procedures must be developed and disseminated by the National Medical Emergency Organization. Furthermore, given the benefits of training programs and exercises (drills), it is advised that EMS facilities organize and carry out yearly training programs and exercises that are tailored to the most common hazards and scenarios in their region.

## Data Availability

The datasets that were used and/or analyzed during the current study are available from the corresponding author upon reasonable request.

## Abbreviations

EMS: Emergency Medical Services
UNISDR: United Nations Office for Disaster Risk Reduction
DRM: Disaster Risk Management
EOP: Emergency Operation Plan
